# Atypical diagnosis of myiasis

**DOI:** 10.48327/mtsi.v3i2.2023.370

**Published:** 2023-05-11

**Authors:** Moreno DUTTO, Carla ZAVATTERO, Elio VINAI, Giuseppe LAURIA, Stefano VANIN

**Affiliations:** 1 Former Medical Entomology and Zoology Consultant, Santa Croce e Carle General Hospital, Cuneo, Italy; 2 Division Pathological Anatomy, Regina Montis Regalis Hospital, Mondovì, Italy; 3 Biomedic Laboratory, Regina Montis Regalis Hospital, Mondovì, Italy; 4 Department Emergency and Acceptance, Santa Croce e Carle General Hospital, Cuneo, Italy; 5 Department of Earth, Environmental and Life Sciences, University of Genova, Genova, Italy

**Keywords:** Myiase, *Lucilia sericata*, Pansement, Soins aux patients, Hygiène, Cuneo, Italie, Europe, Myiasis, *Lucilia sericata*, Dressing, Patient's care, Hygiene, Cuneo, Italy, Europe

## Abstract

Des larves muscoïdes ont été observées sur du matériel de pansement réalisé en automédication, chargé de matières purulentes et provenant d'une plaie nécrotique chez un patient de 91 ans. Ces larves ont été identifiées comme appartenant à l'espèce *Lucilia sericata.* Aucune larve n'a été trouvée dans les tissus du patient. L'observation de larves sur des pansements ne doit pas conduire automatiquement au diagnostic de myiase cutanée. Les mesures d'hygiène et de soins doivent pouvoir éviter leur apparition. Leur risque est plus élevé dans un environnement chaud et avec des moyens limités de soins.

## Abstract (en)

Muscoid larvae were observed on self-medicated dressing material loaded with purulent material taken from a 91-year-old hospital patient. These larvae were identified as *Lucilia sericata.* However, no larvae were found in the patient's tissues. The observation of larvae on dressings should not automatically lead to a diagnosis of cutaneous myiasis.

Myiases are parasitic infestations of living vertebrates, humans included, caused by dipteran larvae belonging to different families (e.g. Calliphoridae, Sarcophagidae, Oestridae, Syrphidae, Piophilidae, etc.) [7]. In Europe, the majority of human myiases are related to the infestation of necrotic lesions or of anatomic cavities with accumulation of excretions/secretions and are mainly due to the larvae of semi-specific myiasigenic species in the genera *Lucilia* Robineau-Desvoidy, 1830, *Calliphora* Robineau-Desvoidy, 1830 (Diptera: Calliphoridae) and *Sarcophaga* Meigen, 1826 (Diptera: Sarcophagidae) [3]. In addition, from a veterinary point of view it is worth mentioning some zoonotic infestations in areas with well-established sheep farming caused by the specific myiasigenic species *Oestrus ovis* (Linnaeus, 1758) (Diptera: Oestridae) [10].

In Southern European countries, another specific myiasigenic species with human medical potential interest is *Wohlfahrtia magnifica* (Schiner, 1862) (Diptera: Sarcophagidae); this species is occasionally responsible for cutaneous (post-traumatic) [5] and cavitary myiasis [11] with the capacity of developping also in healthy tissues. In Italy a few cases of *W. magnifica* myiasis were reported in humans on necrotic lesion [8] and furuncular lesion [2].

In tropical regions fly larvae infestations of lesions and chronic pathologies have a higher incidence, particularly in poor areas and in subjects who live in unhygienic environments [6, 12].

The diagnosis of cutaneous myiasis affecting chronic injuries is quite easy and based on the observation of the maggots in the infested anatomical area [3]. The clinical course of semi-specific myiases is usually favorable and in some cases the presence of larvae feeding on the necrotic tissues can be beneficial, removing the bacteria growing on the lesion [9]. Treatment generally involves mechanical removal of the larvae associated with more frequent replacement of dressings [1, 13].

In August 2019 the laboratory of the Hospital of Mondovì (North-Western Italy), received part of a strip of elastic gauze soaked in purulent exudate with several muscoid larvae (Fig. 1). The gauze was part of an auto-medication of a necrotic lesion associated with microcirculation alteration in the foot of a 91-year-old diabetic man (Fig. 1). The patient was taken to the care service for chronic skin wounds management, where the wound was cleaned and where a new dressing was applied. He was then sent home pending microbiological swab results.

**Figure 1:**
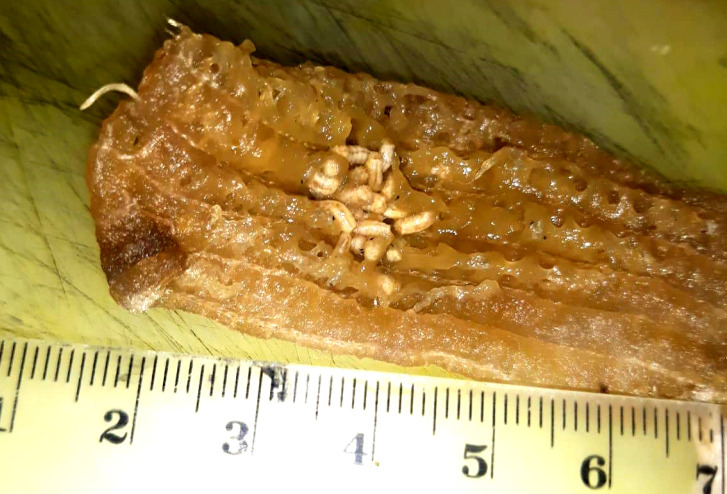
Bandage infested with larvae (photo credit: Vinai E) Bandage infesté de larves (crédit photo : Vinai E)

Larvae were identified, based on morphological diagnostic features – cephalopharyngeal sclerites, respiratory spiracles and intersegmental spines – as *Lucilia sericata* (Meigen, 1826) (Diptera: Calliphoridae) (Fig. 2). Larvae were at the third instar (L3) with an average length, after standard fixation, of 10.3±1.2 mm (N=9).

**Figure 2:**
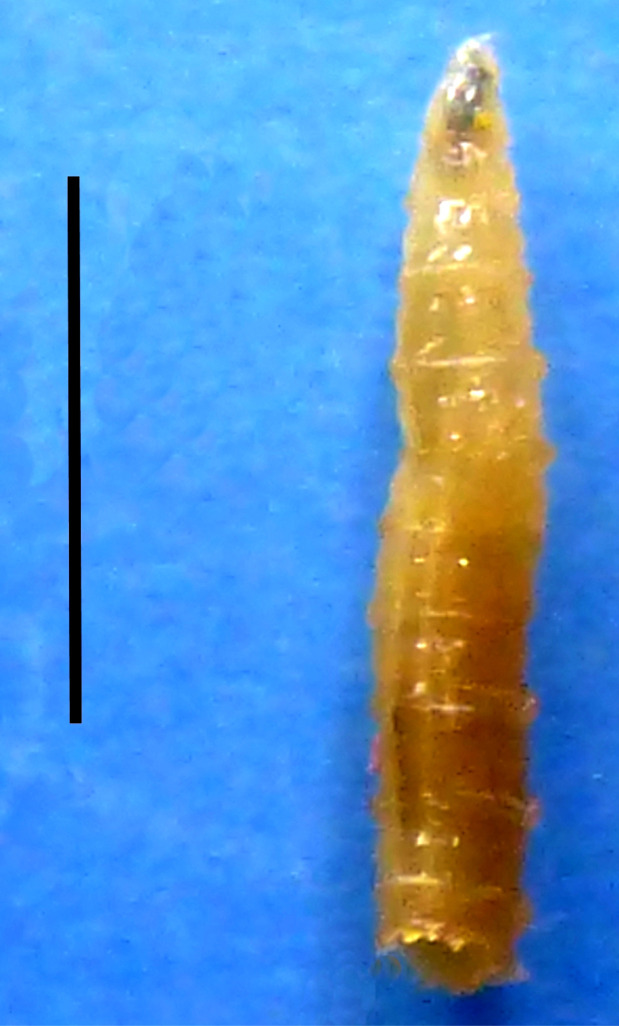
*Lucilia sericata* larva (photo credit: Dutto M.) Scale bar = 5 mm) *Larve de* Lucilia sericata *(crédit photo : Dutto M. Échelle = 5 mm)*

The time of colonization (ToC) was estimated using the data published by Grassberger *et al.* [4], the larval size and the tissue temperature, estimated in the range of 28-34°C using superficial temperature measures. Based on this approach, the ToC was calculated in 36-45 hrs before the delivery to the laboratory (the effect of the change in temperature occurred in the time between the gauze removal and the laboratory delivery can be considered negligible).

The absence of larvae on the patient's tissues, and their exclusive presence on the gauze can be due to the high quantity of purulent organic matter in the dressing enough for the larval feeding. In this case, the presence of larvae on the bandages has to be analysed with caution and cannot be defined as cutaneous myiasis. In fact, in case of inadequate changes of the bandages, especially in association with high exuding flesh, there is an infestation of the bandages not affecting the patient's tissues.

The observations performed in this case allow to draw, from a practical and medico-legal point of view:

1) bandages do not represent a protection to avoid colonization by fly larvae in particular if wet and dirty [6], while indeed they can be a predisposing factor as a source of attraction. In addition to bandages in order to reduce the risk of insect infestation, especially in healthcare facilities, environmental preventive measures have to be considered and implemented such as: presence of UVA trap-lamps, windows equipped with mosquito nets and doors with vertical air flow;
2) the presence of larvae on bandages cannot be used as evidence of a suspect myiasis if the fabrics before their collection and analysis have been stored for a long time in places such as waste bins or room accessible to sarco-saprophagous flies. In case of suspect myiasis the material has to be properly collected and fixated, better by freezing in order to exclude any later infestation that could compromise the quality of the evidence and its interpretation.

In Europe and developed countries, the infestation of dressings represents an occurrence that affects subjects in precarious hygienic conditions. In developing countries, the colonization of bandages and more generally the infestation of wounds or areas affected by chronic pathologies show a higher incidence and can also be associated with the difficulty of accessing health services [12].

## Conflicts of Interest

All authors declare the absence of conflicts of interest.

## Contribution of the Authors

All authors contributed equally to the case study analysis and have read and approved the final manuscript.
